# Synthesis of surfactant-assisted nickel ferrite nanoparticles (NFNPs@surfactant) to amplify their application as an advanced electrode material for high-performance supercapacitors

**DOI:** 10.1039/d4ra02135e

**Published:** 2024-06-25

**Authors:** Muhammad Waheed Mushtaq, Muhammad Shahbaz, Rabia Naeem, Shahid Bashir, Shahzad Sharif, Kainat Ali, Naveed Aslam Dogar

**Affiliations:** a Department of Chemistry, Govt. Graduate College of Science Wahdat Road Lahore Pakistan waheedjaami@gmail.com; b Materials Chemistry Laboratory, Department of Chemistry, GC University Lahore Pakistan mssharif@gcu.edu.pk; c Department of Chemistry, University of Malaya Kuala Lumpur Malaysia

## Abstract

Nickel ferrite nanoparticles (NFNPs) were synthesized in an alkaline medium (pH ∼ 11) using a wet chemical co-precipitation technique. To probe the effect of surfactants on the surface morphology, particle size and size distribution of nanoparticles; two surfactants, namely, cetyl trimethyl ammonium bromide (CTAB) and sodium dodecyl sulphate (SDS), were applied. The native and surfactant-assisted nickel ferrite NPs were characterized using Fourier transform infrared (FTIR) spectroscopy, X-ray diffraction (XRD), scanning electron microscopy (SEM), atomic force microscopy (AFM), dynamic light scattering (DLS) and transmission electron microscopy (TEM). The addition of surfactants (CTAB/SDS) effectively controlled the secondary growth of nickel ferrite particles and reduced their size, as examined by XRD, AFM, DLS, SEM and TEM. Characterization technique results affirmed that CTAB is a more optimistic surfactant to control the clustering, dispersion and particle size (∼22 nm) of NFNPs. To identify the impact of ferrite particle size on charge storage devices, their electrochemical properties were studied by using cyclic voltammetry (CV), galvanic charge–discharge (GCD) and electrochemical impedance spectroscopy (EIS) in 1 M KOH electrolyte through three-electrode assembly. NiFe_2_O_4_@CTAB showed a specific capacity of 267.1 C g^−1^, specific capacitance of 593.6 F g^−1^ and energy density of 16.69 W h kg^−1^, which was far better than the performances of other synthesized native NFNPs and NiFe_2_O_4_@SDS having larger surface areas.

## Abstract 2

Nickel ferrite nanoparticles (NFNPs) were synthesized in an alkaline medium (pH ∼ 11) using a wet chemical co-precipitation technique.

## Graphical abstract

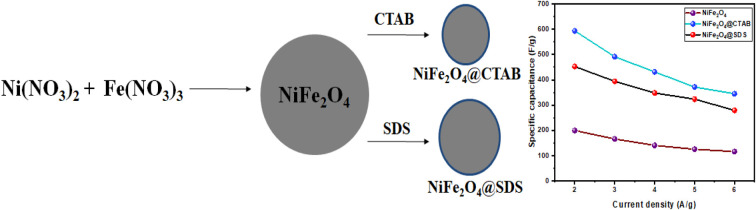

## 1. Introduction

Over the past decades, there has been a growing focus on the preparation and characterization of nanomaterials exhibiting distinct physical and chemical properties. The main reason for heightened interest in nanomaterials is primarily attributed to particle size, surface area and topography in the field of nanotechnology.^1^ From the class of nanomaterials, NiFe_2_O_4_ nanoparticles have attracted great interest for their peculiar applications in different fields. They have a vast area of utilization in the fields of ferrofluids, magnetic materials, catalysts, electronic and microwave devices, information storage and energy storage.^2,3^ NiFe_2_O_4_ nanoparticles play a vital role as catalysts in a number of significant reactions including oxidative dehydrogenation of hydrocarbons, alcohol decomposition, selective oxidation of carbon monoxide and hydrogen peroxide decomposition.^4^ In biological systems, NiFe_2_O_4_ nanoparticles have been used for drug delivery where the conventional methodology fails to do the required task. Moreover, the use of NPs in magnetic resonance imaging (MRI), magnetic hyperthermia, and bio as well as chemosensors^5^ is reported.

Spinel phase nanoparticles have potential applications in the field of energy storage devices. The use of NiFe_2_O_4_ nanoparticles as the electrode material in lithium-ion batteries can be proved effective for superior electrical conductivity, specific capacity, enhanced electrochemical reactivity and more importantly the lower cost for energy production. NiFe_2_O_4_ nanoparticles possess distinct properties such as high adsorption capacity, marvelous stability, safety, good magnetic properties and electronic tunability that make them a good candidate to remove environmental pollutants by the dint of their property of adsorption. Spinel nanoparticles are also used as sensors for the detection of trace pollutants even at lower concentrations with high sensitivity and excellent selectivity.^6^ As NiFe_2_O_4_ nanoparticles are environmentally friendly and abundant in nature, various researchers have studied their electrochemical performance.

Previously reported NiFe_2_O_4_ nanoparticles showed a specific capacitance of 120 F g^−1^.^7^ Kumar *et al.* synthesized mesoporous NiFe_2_O_4_ nanoparticles *via* the hydrothermal method and studied the effect of the surface area of the nanoparticles on electrochemical response in a three-electrode assembly.^8^ Saom *et al.* observed a specific capacitance of 207 F g^−1^ in 1 M Na_2_SO_4_ electrolyte.^9^ Geo *et al.* also reported that the larger surface area improved the performance of the nanoparticles showing a specific capacitance of 204.9 F g^−1^ at the current density of 1 A g^−1^.^10^ Furthermore, Arun *et al.* inferred that the specific capacitance of 277 F g^−1^ retained up to 5000 cycles by decreasing the particle size of the NiFe_2_O_4_ nanoparticles.^11^ It is evident from the study that morphology is one of the primary factors that can improve the electrochemical properties of the materials.^12,13^ Thus, the researchers have been trying to synthesize electrode materials with large surface areas to enhance the electrochemical performance of energy storage devices. Hu *et.al.* reported organic materials showing elemental stability as well as functional tenability for use as cathodes for energy storage devices, exhibiting significant electrochemical performance.^14^

There are several unique methods for the syntheses of NiFe_2_O_4_ nanoparticles, including solid state reaction method,^15^ reverse micelle method, sol–gel method,^16^ hydrothermal techniques,^17^ shock wave method,^18^ aerosolization,^19^ microwave hydrothermal method,^20^ ultra-sonic hydrothermal method,^21^ sonochemistry^22^ and ball milling method.^23^ The basic drawbacks of these techniques include the precision in maintaining the particle size and distribution. In the presence of atmospheric oxygen, oxidation of the particles is shielded to prevent agglomeration of NiFe_2_O_4_ nanoparticles by coating them with special kinds of surfactants like sodium dodecyl sulphate and oleic acid.^24^ The co-precipitation method has a peculiar advantage over all other methods for the synthesis of nanoparticles in that it does not require additional microwave heating and it is easy to control the size and size distribution.^5^

In this study, the co-precipitation technique was used to synthesize NiFe_2_O_4_ nanoparticles. The particle size of the nanoparticles was tuned by using surfactants such as cetyltrimethyl ammonium bromide (CTAB) and sodium dodecy benzene sulphonate (SDS). X-ray diffraction (XRD), Fourier transform infrared (FTIR), scanning electron microscopy (SEM), and atomic force microscopy (AFM). The electrochemical properties of the nanoparticles were studied by employing different electroanalytical techniques such as cyclic voltammetry (CV), galvanic charge–discharge (GCD) and electrochemical impedance spectroscopy (EIS), which confirmed that the NiFe_2_O_4_@CTAB, having smaller particle size coupled with larger surface area can be used as potential electrode material for futuristic energy storage devices.

## 2. Materials and methods

### 2.1. Chemical synthesis

#### 2.1.1. Synthesis of nickel ferrite (NiFe_2_O_4_) nanoparticles

Nickel ferrite NPs were synthesized using a modified wet chemical co-precipitation method. 0.4 M ferric nitrates and 0.2 M nickel nitrate solutions in equal volume ratio were mixed in a three-neck round bottom flask. The initial pH of the mixed binary solution was recorded as 1.34. To initiate the chemical reaction, 3 M NaOH (as precipitant) was added dropwise in the mixed solution with constant stirring to achieve a pH close to 11. The grayish-black residue of ferrite NPs appeared in the reaction mixture which was heated up to 85 °C for 90 minutes. The solid nickel ferrite NPs were washed many times with distilled water and ethanol and dried in an oven at 65 °C for 48 hours.
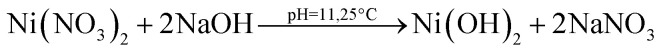

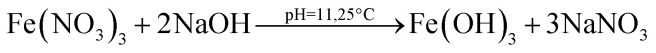

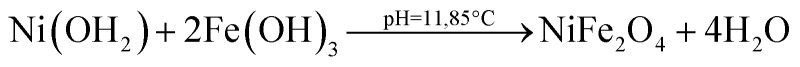


#### 2.1.2. Synthesis of nickel ferrite NPs with the assistance of surfactants

To examine the effect of surfactants on the synthesis of nickel ferrite NPs, 8–10 drops of 0.04 M solution of surfactants, cetyltrimethylammonium bromide (CTAB), or sodium dodecylbenzene sulphonate (SDS) were added during co-precipitation. The temperature of the reaction mixture was increased to 80 °C for 90 minutes. The resultant black residue was washed with distilled water and ethanol to remove excess ions (nitrate and sodium) and the product was dried in an oven at 65 °C for two days. The surfactant-assisted prepared nickel ferrite products were labeled as NFNPs@CTAB (with cetyltrimethylammonium bromide) and NFNPs@SDS (with sodium dodecylbenzene sulphonate) for further characterization and electrochemical application.

### 2.2. Characterization

#### 2.2.1. Crystallographic study of nickel ferrite (NiFe_2_O_4_)

**Fig. 1:**
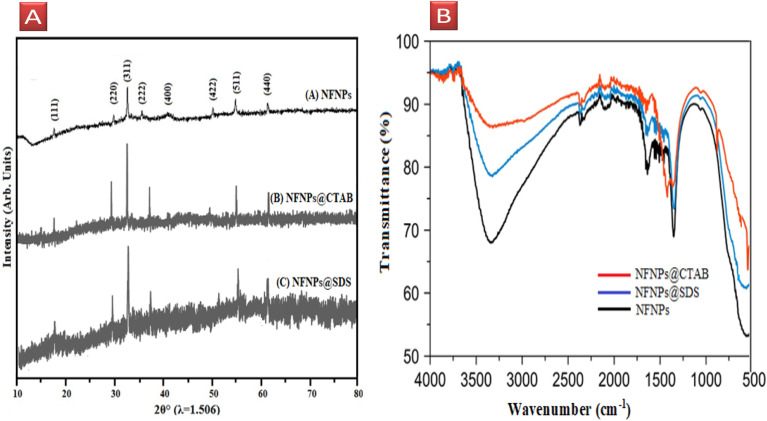
(A) XRD patterns and (B) FT-IR spectrum of native NiFe_2_O_4_ and surfactant-assisted NiFe_2_O_4_@CTAB, NiFe_2_O_4_@SDS nanoparticles.

X-ray diffraction (XRD) technique was used to identify the effects of cationic and anionic capping agents on the surface morphology of nickel ferrite nanoparticles (NFNPs). XRD patterns of pure and surfactant-assisted NFNPs are shown in Fig. 1A. All the diffraction peaks are in correspondence with diffraction angles at 17.99°, 30.10°, 34.94°, 37.01°, 42.86°, 54.05°, 57.01° and 61.89° and are parallel with JCPDS card no (22-1086) ratifying the cubic inverse spinel phase structure of native NFNPs. The space group symmetry *Fd*3*m*, *O*_h_ and powder crystalline morphology of pure nickel ferrite NPs showed their high purity index.^24,25^ Sharp XRD peaks at (111), (220), (311), (222), (400), (422), (511) and (440) are also justifying the crystalline index of NFNPs. The average size of pure nickel ferrite nano-crystals was 46 nm as measured from widely distributed diffraction peak (311) using Debye Sherrer eqn (1).

1 *D* = 0.9*λ*/*β *cos* θ*

where *D* is the crystal diameter, *λ* is the wavelength of CuKα X-ray, *β* is FWMH diffracted rays and *θ* is the Bragg's angle.^26^

The addition of surfactants has a significant impact on the regular arrangement of the particles on crystal growth as well as the size of NFNPs. The presence of a long alkyl hydrocarbon chain in both surfactants acts as an impurity and induces an amorphous nature in regular lattice points of ferrite nanoparticles. The presence of surfactants serves as a capping agent, effectively minimizing the particle size of nickel ferrite nanoparticles (NFNPs) to approximately 22 nm and 27 nm with NFNPs@CTAB and NFNPs@SDS, respectively. This size reduction was determined using the Debye–Sherrer equation. In the case of CTAB and SDS, the broadening of the diffraction peak (311) indicates the impact of capping agents in preventing NP clustering, ultimately resulting in a reduction in the particle size. Huang *et al.* also observed diffraction peak broadening at (311) when ferrite particle size was decreased to below 20 nm.^27^ Nickel ferrite NPs prepared using a cationic surfactant (NFNPs@CTAB) have a pronounced effect on size reduction as compared to those prepared using the anionic surfactant. The adsorbed hydroxide ions on the surface of ferrite particles promote the coating of cationic surfactants and enhance their dispersion by reducing agglomeration. So, cationic surfactants are more capable of dispersing and decreasing particle size of nickel ferrite NPs as compared to the anionic one.

#### 2.2.2. FTIR spectrum of pure and surfactant-assisted nickel ferrite NPs

To identify the distinctive vibrations of metal ions within their lattice structures, a Fourier transform-infrared (FTIR) spectrum was examined within the range of 4000–500 cm^−1^. The FTIR spectra of both pure as well as surfactant-enhanced nickel ferrite nanoparticles (NPs) are depicted in Fig. 1B. The broad absorption band between 3200–3600 cm^−1^ indicates the presence of moisture content in the samples and the vibrations of water molecules –OH groups absorbed on the ferrite NP surface. The presence of a characteristic metal–oxygen peak (600–500 cm^−1^) is evident in all IR spectra, confirming the precise preparation of nickel ferrite in both the absence and presence of surfactants. A spectral peak at 508 cm^−1^ signifies the native stretching vibrations of the metal–oxygen interaction in the metal ferrite at tetrahedral (*T*_h_) and octahedral (*O*_h_) positions.^28^ The peaks at 850 cm^−1^ and 1000–1400 cm^−1^ for the surfactant-coated nickel ferrite NPs indicate the presence of –CH_2_ and C–O, C–H linkages, respectively, within the surfactant (CTAB and SDS) structures.^29,30^

#### 2.2.3. Microscopic studies of pure and surfactant-assisted nickel ferrite NPs

**Fig. 2:**
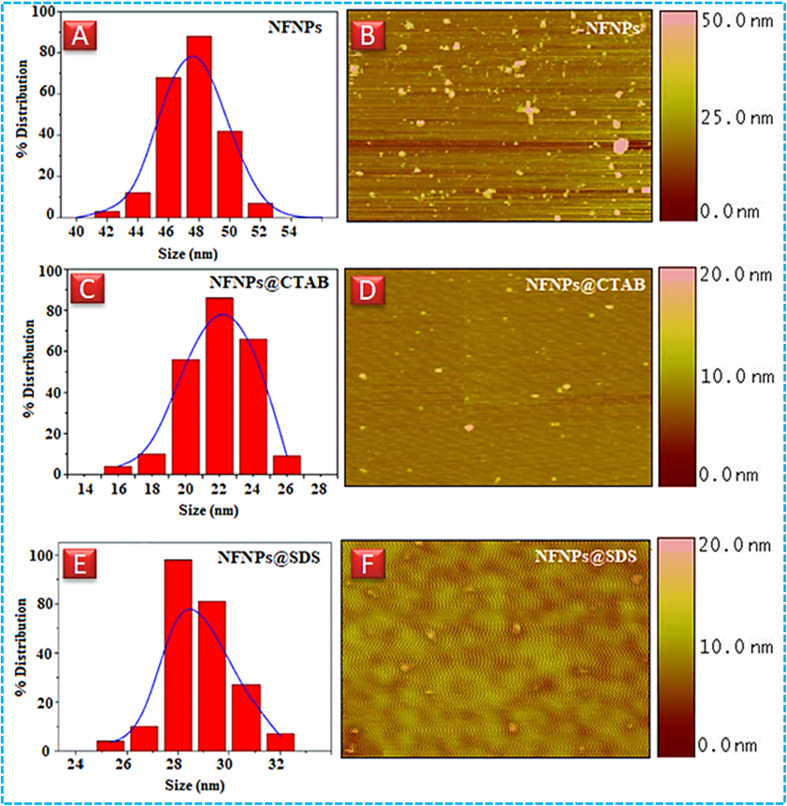
Dynamic light scattering images of (A) NiFe_2_O_4_ (C) NiFe_2_O_4_@CTAB (E) NiFe_2_O_4_@SDS, atomic force microscopy images (B) NiFe_2_O_4_ (D) NiFe_2_O_4_@CTAB, and (F) NiFe_2_O_4_@SDS.

**Fig. 3:**
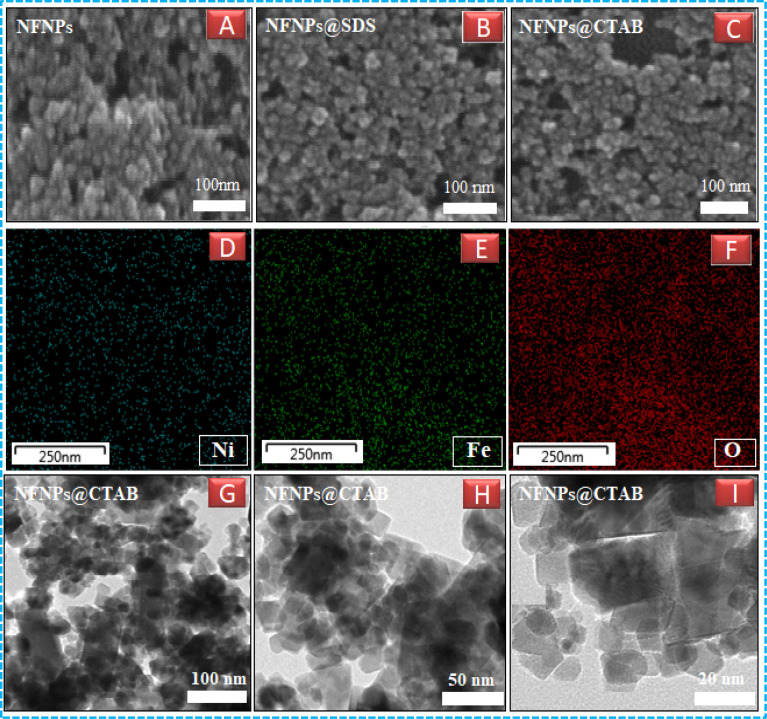
Scanning electron microscope images of (A) NiFe_2_O_4_ (B) NiFe_2_O_4_@SDS (C) NiFe_2_O_4_@CTAB, Elemental mapping micrographs (D) nickel-Kα (E) iron-Kα (F) oxygen-Kα, Transmission electron microscope images of NiFe_2_O_4_@CTAB at (G) 100 nm (H) 50 nm, and (I) 20 nm.

To probe the effect of surfactants on the surface topography, morphology and size reduction of nickel ferrite nanoparticles, various microscopic studies including dynamic light scattering, atomic force microscopy, elemental mapping, scanning electron microscopy and transmission electron microscopy were performed and their images are shown in Fig. 2 and Fig. 3. Native nickel ferrite NPs having the size around ≈ 45 nm are spherical in shape and suffering from agglomeration effect as shown in AFM images. However, with the addition of CTAB and SDS, the stabilization of the ferrite particles enhanced with the decrease of nucleation growth as explained in the literature also.^31^ The addition of the surfactants promotes the uniform distribution of the particles as shown in AFM, SEM and TEM images. Nickel ferrite NPs obtained using CTAB, are more uniformly distributed and reduced in their hydrodynamic size as compared to SDS. In fact, the nature of surfactants has a pronounced effect on reducing the particle size. The alkaline media of the reaction makes the particle negatively charged due to the adsorption of the hydroxyl groups (–OH). Thus, the cationic capping agents such as CTAB are more effectively adsorbed at the surface of the particles to control their nucleation and growth, also called Ostwald's ripening. TEM studies of CTAB-coated nickel ferrite NPs (as shown in Fig. 3) were also performed to obtain a more precise idea about the role of CTAB on the surface morphology and controlled growth of nickel ferrite NPs. TEM images reveal spherical morphology of NFNPs as also confirmed by AFM and SEM. The average particle size (21 nm) as determined by TEM is very close to XRD calculations. The coagulation of ferrite NPs was also reduced by cationic CTAB coating as shown in Fig. 3G–I. Elemental mapping images confirm the presence of Ni, Fe and oxygen in the nickel ferrite particles as shown in Fig. 3D–F. The nanoparticle size range of native and surfactant-assisted synthesized nickel ferrite NPs measured by various techniques is shown in Table 1.

**Table 1:** Range of the particle size measured by various characterization techniques

Sample	TEM	DLS (nm)	PDI XRD (nm)	AFM (nm)
NiFe_2_O_4_	—	48	0.28 45	50
NiFe_2_O_4_@CTAB	21	24	0.11 22	20
NiFe_2_O_4_@SDS	—	28	0.19 27	30

### 2.3. Electrochemical analysis

Electrochemical analysis was performed on OrigaLys , France electrochemical work station OrigaFlex-OGF+01A, OGF+05A and OrigaFlex-OGFEIS using a three-electrode assembly with nickel ferrite NPs as the working electrode, platinum wire as counter electrode and silver–silver chloride (Ag/AgCl) as the reference electrode. Electrochemical studies were conducted in 1 M KOH solution as an electrolyte. NFNPs working electrode was prepared by grinding the sample to a fine powder. Nickel foam was used as a substrate material to deposit the nickel ferrite NPs. Prior to the deposition of the active material on NF, it was treated sequentially with 3 M HCl, acetone and ethanol. NFNPs, polyvinylidene fluoride (PVDF) binder and activated carbon (80 : 10 : 10) were mixed in *N*-methyl pyrrolidone (NMP) and stirred for 6 hours to prepare a homogeneous slurry. The drop casting method was employed to deposit the slurry onto the pre-treated NF and was kept for drying in an oven at 40 °C for 4 hours.

#### 2.3.1. Cyclic voltammetry

**Fig. 4:**
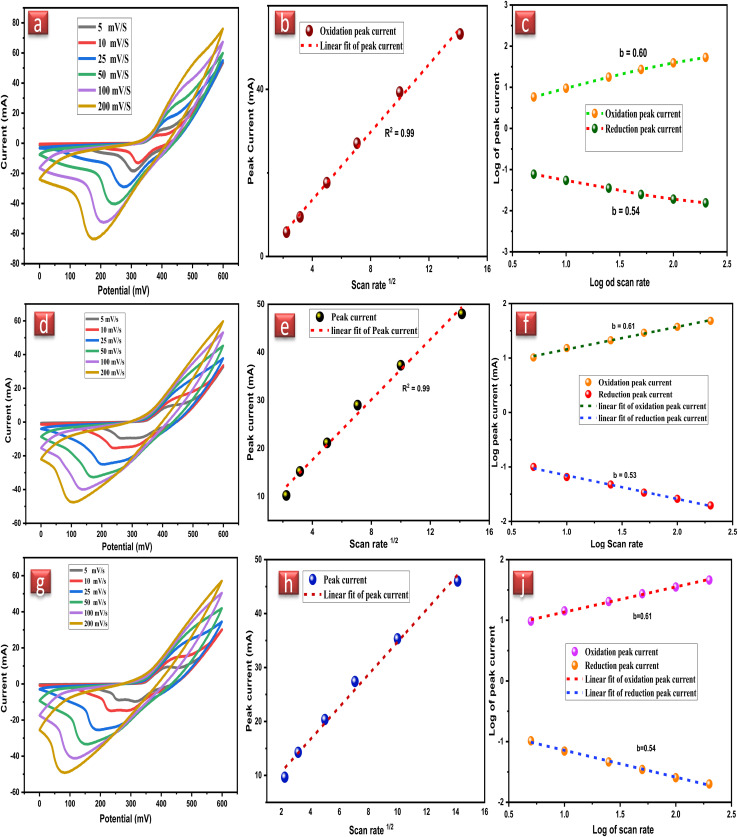
(a) CV plot of native NiFe_2_O_4_ NPs (b) linear regression coefficient of NiFe_2_O_4_ NPs (c) *b* values of NiFe_2_O_4_ NPs (d) CV plot of NiFe_2_O_4_@CTAB (e) linear regression coefficient of NiFe_2_O_4_@CTAB (f) *b*-values of NiFe_2_O_4_@CTAB (g) CV plot of NiFe_2_O_4_@SDS (h) linear regression coefficient of NiFe_2_O_4_@SDS, and (i) *b*-values of NiFe_2_O_4_@SDS.

Electrochemical measurements were taken by selecting the potential window ranging from 0 mV to 600 mV. Cyclic voltammetry was carried out at various scan rates (5, 10, 25, 50, 100, and 200 mV s^−1^) as shown in Fig. 4a. Oxidation and reduction peaks at negative and positive scans vividly depicted the existence of the redox mechanism. In nickel ferrites, the conductivity was enhanced due to the interconversion of electrons between Fe^2+^/Fe^3+^ and Ni^2+^/Ni^+^. A mathematical approach was used to discuss the faradaic nature of NiFe_2_O_4_ NPs and a graph was plotted between the peak currents and square root of the scan rate Fig. 4b. The graphs have a regression coefficient of approximately equal to 1 that elaborated the presence of reversible redox reaction in NFNPs. Furthermore, power law (eqn (2)) was employed to study the relationship between peak oxidation peak current and reduction peak current *versus* the scan rate.^32^

2 *i*_p_ = *aυ*^*b*^

where ‘*a*’ and ‘*b*’ are the variables, *i*_p_ is the peak current and *υ* is the value of the scan rate. The plotted graph gave a straight line and the slope of the graph showed the *b*-value Fig. 4c. The *b*-value has significance in evaluating the capacitive and battery-like nature of the material. If the *b*-value is near 1, it signifies the capacitive nature of the electrode, while on the other hand, the value of *b* closer to 0.5 denotes the diffusive nature of electrode material.^25^

CV plot remained the same at all the scan rates but only differed in the peak current after increasing the scan rate, which displays that the native NiFe_2_O_4_ NPs working electrodes have excellent rate capability. Retention of the CV peak shape also signifies the electrochemical reversibility of the NiFe_2_O_4_ NPs working electrode signifying the fast diffusion of charges between the electrode and electrolyte. The stability of NiFe_2_O_4_ NPs can also be estimated by the linear increase in redox peak by increasing scan rate and electrochemical active components in NiFe_2_O_4_ NPs enhance the conductivity of the electrode.

#### 2.3.2. Galvanostatic charge–discharge

**Fig. 5:**
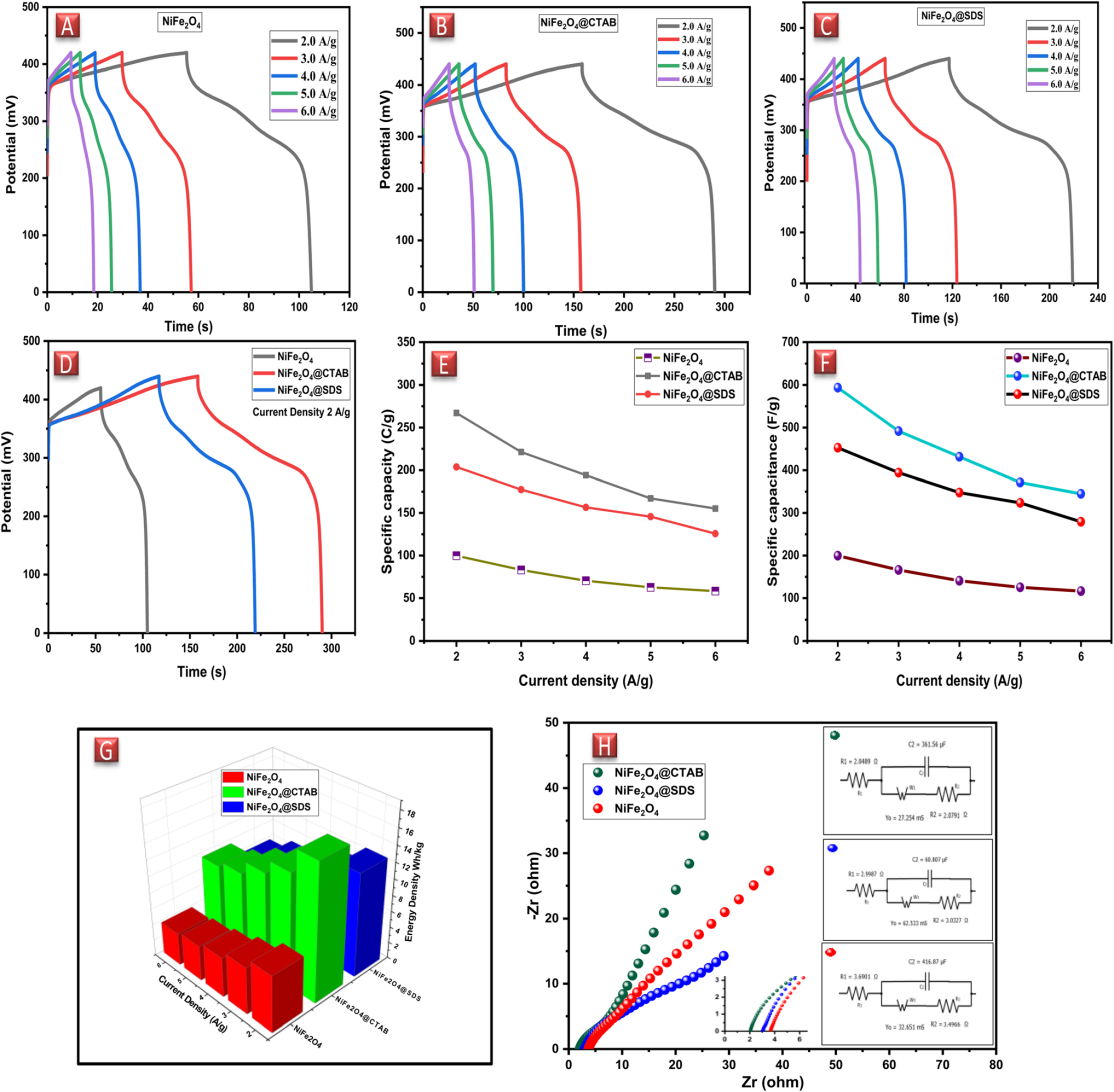
(A) GCD measurements of NiFe_2_O_4_, (B) NiFe_2_O_4_@CTAB and (C) NiFe_2_O_4_@SDS. (D) Comparison of GCD curves, (E) specific capacities, (F) specific capacitance (G) bar-chart showing energy densities and (H) EIS plots of NiFe_2_O_4_, NiFe_2_O_4_@CTAB and NiFe_2_O_4_@SDS.

To investigate the charge capacity of NiFe_2_O_4_ nanoparticles, galvanostatic charge–discharge measurements were taken at discrete current densities (2, 3, 4, 5 and 6 A g^−1^). In correspondence with the cyclic voltammetric measurements, the plateaus in GCD curves also showed the pseudocapacitive nature of NiFe_2_O_4_ NPs. In charging curves, the current increased abruptly due to the capacitive nature that was followed by plateaus, which confirmed that the current contribution was changed as a result of the diffusive-controlled mechanism (Fig. 5A–C). The NiFe_2_O_4_ NPs hold excellent reaction reversibility that could be confirmed by the presence of symmetrical plateaus in the discharge curves. Specific capacities of native and surfactant-assisted NFNPs (NiFe_2_O_4_@CTAB and NiFe_2_O_4_@SDS) were determined at different current densities using eqn (3), showing maximum specific capacities of 99.8, 267.1 and 203.7 C g^−1^, respectively, at a current density of 2 A g^−1^, which decreased with increasing current density (Fig. 5E).^33^

The specific capacitances of the NFNPs were also determined using eqn (4), which were 199.6, 593.6 and 452.3 F g^−1^ for native NiFe_2_O_4_, NiFe_2_O_4_@CTAB and NiFe_2_O_4_@SDS, respectively, at a current density of 2 A g^−1^, which decreased with increasing current density (Fig. 5F).^26^3
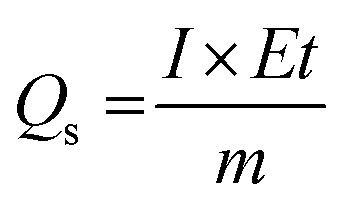
4
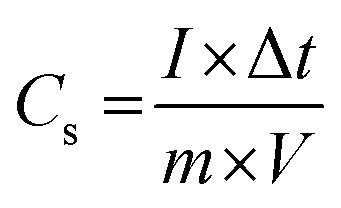
Here, the expression *I*/*m* indicates the current density and Δ*t* signifies the discharge time of the GCD curve. From the specific capacity values determined from GCD analysis, energy density and power density were calculated by using eqn (5) and (6).^34^5
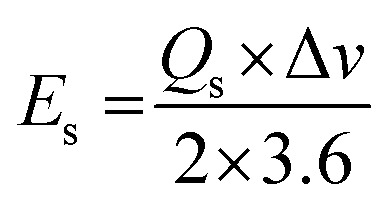
6
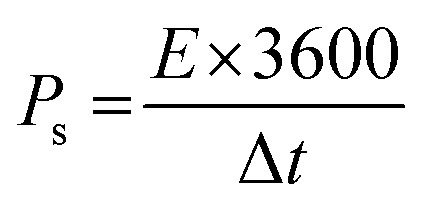


Native NFNPs, NiFe_2_O_4_@CTAB and NiFe_2_O_4_@SDS showed the maximum energy density of 6.93, 16.69 and 12.73 W h kg^−1^ and power densities of 1200, 1420 and 1350 W kg^−1^, respectively, at a current density of 2 A g^−1^. A comparison of electrochemical parameters of the synthesized native NiFe_2_O_4_, NiFe_2_O_4_@CTAB and NiFe_2_O_4_@SDS is shown in Table 2.

**Table 2:** Comparison of specific capacities, specific capacitances, energy densities and power densities of NFNPS

Sample	Specific capacity (C g^−1^) at 2 A g^−1^	Specific capacitance (F g^−1^) at 2 A g^−1^	Energy density (W h kg^−1^) 2 A g^−1^	Power density (W h kg^−1^) at 6 A g^−1^
NiFe_2_O_4_	99.8	199.6	6.93	1200
NiFe_2_O_4_@CTAB	267.1	593.6	16.69	1420
NiFe_2_O_4_@SDS	203.7	452.3	12.73	1350

#### 2.3.3. Electrochemical impedance spectroscopy

To evaluate the inclusive conductivity of NiFe_2_O_4_ NPs electrode electrochemical impedance spectroscopy was employed. EIS was carried out in an AC frequency range of 0.1 Hz to 100 KHz and Nyquist plots of three samples were taken (Fig. 5H). The EIS spectrum showed that there was small equivalent solution resistance (*E*_SR_) due to ions diffusion through the electrolyte and the interface resistance of the electrode. Moreover, in the domain of higher frequencies, the presence of semicircle indicated the charge transfer resistance (*R*_ct_), which can be obtained from equivalent circuits.^14,27^ Charge transfer resistance also known as Faraday's resistance is faced by electrochemical reactions on the interface of the NiFe_2_O_4_ NPs electrode.

In the case of the NiFe_2_O_4_ NP electrode, the values of *E*_SR_ and *R*_ct_ were recorded as 3.69 Ω and 3.49 Ω, respectively. The straight line signified the Warburg impedance indicating ion diffusion and pseudocapacitive behavior of the electrode material (in red). NiFe_2_O_4_@SDS exhibited an *E*_SR_ of 2.99 Ω with an *R*_ct_ of 3.03 Ω (in blue). While NiFe_2_O_4_@CTAB indicated an *E*_SR_ value of 2.05 Ω with a small semicircle showing the negligible *R*_ct_ of 2.08 Ω, which advocated the positive effect of CTAB on the NiFe_2_O_4_ NPs (in green). The presence of Warburg resistance also proved the pseudocapacitive nature of the electrode material.

## 3. Conclusions

Herein, we have reported the synthesis of nickel ferrite nanoparticles by controlling surface area utilizing different surfactants; cationic surfactant (CTAB) and anionic surfactant (SDS). Different structural analysis techniques confirmed that CTAB proved a more promising tool to control surface topography, morphology, particle size and dispersion of nickel ferrite NPs. Electrochemical techniques such as CV, GCD and EIS were used to evaluate the electrochemical performance of the synthesized materials. Native NFNPs, NiFe_2_O_4_@CTAB and NiFe_2_O_4_@SDS showed specific capacity as 99.8, 267.1, 203.7 C g^−1^, specific capacitance as 199.6, 593.6, 452.3 F g^−1^, energy density of 6.93, 16.69, 12.73 W h kg^−1^ and power density of 1200, 1420 and 1350 W kg^−1^, respectively. The results illustrated that the reduced particle size coupled with a larger surface area of NFNPs@CTAB enhanced the electrochemical performance of nickel ferrite nanoparticles as a potential candidate for electrode material in the field of electrochemical energy storage devices.

## Author contributions

Muhammad Waheed wrote the manuscript. Shahbaz performed the electrochemical analysis. Rabia performed the experiments. Shahid critically proofed the manuscript. Shahzad designed the study, Kainat performed the synthesis and Naveed prepared the graphs.

## Conflicts of interest

The authors declare that they have no known competing financial interests or personal relationships that could have appeared to influence the work reported in this paper.
